# 化疗对肺癌患者生活质量及焦虑情绪的影响及分析

**DOI:** 10.3779/j.issn.1009-3419.2012.08.03

**Published:** 2012-08-20

**Authors:** 淑芳 李, 燕 王, 士珍 辛, 建存 曹

**Affiliations:** 1 300070 天津，天津医科大学研究生院 Graduate School, Tianjin Medical University, Tianjin 300070, China; 2 300052 天津，天津医科大学总医院肿瘤内科 Depertment of Oncology, Tianjin Medical University General Hospital, 300052 Tianjin, China

**Keywords:** 肺肿瘤, 化疗, 生活质量, 焦虑, Lung neoplasms, Chemotherapy, Quality of life, Anxiety

## Abstract

**背景与目的:**

通过观察肺癌患者化疗前后生活质量及焦虑情绪的改变，探讨化疗对肺癌患者生活质量及焦虑情绪的影响。

**方法:**

随机抽取住院化疗的肺癌患者58例，分别于化疗前、化疗后2周期1星期内、化疗后4周期1星期内评估临床疗效，并进行肺癌患者生活质量量表（QLQ-C30）和ZUNG焦虑自评量表（SAS）评分。

**结果:**

化疗前，生活质量功能领域、疲乏、呼吸困难条目得分较高，有焦虑情绪的占56%，SAS得分为49.54±5.64；焦虑症状与失眠轻度相关（*P* < 0.05）；化疗后2周期，呼吸困难得分下降，失眠、食欲丧失得分上升，较化疗前相比差异有统计学意义（*P* < 0.05）；有焦虑情绪的占80%，SAS得分为52.48±6.10，较化疗前相比差异有统计学意义（*P* < 0.05）；有基础病患者SAS得分高于无基础病患者，差异有统计学意义（*P* < 0.05）；焦虑症状与疲乏、呼吸困难轻度相关（*P* < 0.05）。化疗后4周期，躯体、角色、情绪、社会功能得分下降明显，恶心呕心、食欲不振、便秘、经济困难条目得分上升，较化疗后2周期相比差异有统计学意义（*P* < 0.05）；有焦虑情绪的占72%，SAS得分为54.82±6.55，较化疗后2周期相比无统计学差异；SAS得分与KPS呈负相关（*P* < 0.05）；焦虑症状与疲乏、失眠相关（*P* < 0.01），与便秘轻度相关（*P* < 0.05）。

**结论:**

化疗过程中，部分肺癌患者躯体症状得到缓解，焦虑情绪明显增加，生活质量有所下降，医务工作者应及时评价患者生活质量及情绪改变，提高患者生活质量，积极地对患者进行心理疏导治疗。

## Abstract (en)

**Background and objective:**

This study aims to observe the changes in quality of life as well as the anxiety among lung cancer patients before and after chemotherapy. This work also aims to explore the effect of chemotherapy on quality of life and anxiety.

**Methods:**

Fifty-eight lung cancer patients were evaluated based on clinical outcomes, EORTC QLQ-C30, and on SAS questionnaires before chemotherapy, one week after two courses of chemotherapy, and one week after four courses of chemotherapy.

**Results:**

Before chemotherapy, functioning scale, fatigue, and dyspnoea scores were high, the rate of anxiety was 56%, while the SAS score was 49.54±5.64. Anxiety was found to be positively correlated with insomnia (*P* < 0.05). After two courses of chemotherapy, dyspnoea scores decreased, while insomnia and appetite loss scores increased. The difference was statistically significant (*P* < 0.05). The rate of anxiety was 80%, and the SAS score was 52.48±6.10. The difference was statistically significant compared with that before chemotherapy (*P* < 0.05). The SAS scores of patients with disease history were higher than those of patients without disease history (*P* < 0.05). SAS score was found to be positively correlated with fatigue and dyspnoea (*P* < 0.05). After four courses of chemotherapy, the physical, role, emotional, and social function scores decreased, while the nausea and vomiting, appetite loss, constipation, and financial impact scores increased. The difference was statistically significant (*P* < 0.05). The anxiety rate was 72%, and the SAS score was 54.82±6.55. The difference was statistically significant (*P* < 0.05). The SAS score was negatively correlated with KPS (*P* < 0.05), significantly positively correlated with fatigue and insomnia (*P* < 0.01), and positively correlated with constipation (*P* < 0.05).

**Conclusion:**

A number of patients have experienced symptom relief, but during chemotherapy, the patients had significant anxiety. Thus, quality of life decreased. The quality of life and emotions of lung cancer patients should be evaluated, and positive psychological intervention should be given to improve quality of life.

原发性支气管肺癌，简称肺癌，是一种严重影响患者生存的疾病。2008年全球数据^[1]^显示：肺癌占男性恶性肿瘤发病率和死亡率的第一位，女性分别占第四位和第二位。由于肺癌恶性程度高，并且在诊断时常会出现远处转移，所以化疗是肺癌患者不可缺少的治疗方法，化疗过程中患者不仅要承受癌症诊断结果带来的心理打击，还要承受化疗带来的痛苦和诸多的不适应，情绪反应强烈。本研究通过对肺癌患者化疗前后生活质量及焦虑情绪改变的研究，为医护人员及时发现并改善患者的生活质量和情绪提供依据。

## 1 材料与方法

### 1.1 临床资料

2009年5月-2012年5月前瞻性随机选取我科收治的已确诊的肺癌患者58例。纳入标准：①首次病理确诊的原发性支气管肺癌并未进行过化疗的患者；②根据2009年NCCN肺癌国际分期标准为Ⅲ期-Ⅳ期；③体力评分KPS评分≥70分；④预期生存期 > 3个月；⑤愿意接受问卷调查。排除标准：①既往精神病史；②文盲者或者小学以下文化程度者；③经解释后仍不能理解问卷条目者；④既往接受抗焦虑治疗；⑤既往酒精或药物依赖史。化疗方案：非小细胞肺癌一线化疗予含铂两药方案（铂类+吉西他滨或长春瑞滨、紫杉醇、培美曲塞等），小细胞肺癌一线化疗予依托泊甙或替尼泊苷+顺铂方案，疾病进展给予二线化疗方案（多西紫杉醇、培美曲塞等），其余均继续原方案化疗，化疗每2周期后进行病情评估。

### 1.2 方法

化疗前记录患者的一般资料，包括患者的性别、年龄、BMI、KPS评分、病理类型、肺癌分期、化疗方案、文化程度、有无基础疾病等。分别于化疗前、化疗后2周期1星期内、化疗后4周期1星期内采用EORTC QLQ-C30量表^[2]^及Zung焦虑自评量表^[3]^进行调查，解释并告知回答问卷的方法。

### 1.3 资料收集

研究者对入选患者进行跟踪调查并进行临床疗效的评价。按照RECIST标准，疗效评价分为：①完全缓解（complete response, CR）：所有已知病灶消失并保持最少4周；②部分缓解（partial response, PR）：肿瘤基线病灶长径总和缩小≥30%并保持4周以上；③病灶稳定（stable disease, SD）：基线病灶长径总和有缩小但未达PR或增加但未到PD；④疾病进展（progressive disease, PD）：基线病灶长径总和增加≥20%或出现新病灶。总有效率为PR+CR人数之和所占治疗观察人数的百分率，同时对患者进行EORTC QLQ-C30、SAS量表问卷调查。EORTC QLQ-C30量表：对于功能领域和总体健康状况领域得分（标化分）越高说明功能状况和生命质量越好，对于症状领域得分越高表明症状或问题越多^[2]^。SAS量表：按照中国常模，SAS标准分的分界值为50分，≥50分表明存在焦虑症状。

### 1.4 统计学方法

应用SPSS 17.0统计软件，对化疗不同阶段生活质量、焦虑情绪得分以及焦虑情绪与患者性别、年龄、KPS、生活质量各条目间关系进行分析。得分如果服从正态分布，用Mean±SD表示，采用*t*检验、方差分析和*SNK*两两比较法；如不符合正态分布，用中位数表示，采用*Kruskal-Wallis*秩和检验。对化疗前后SAS得分与生活质量各领域得分进行*Pearson*线性相关分析，以*P* < 0.05为差异有统计学意义。

## 2 结果

### 2.1 一般资料

共向58例患者发放问卷，回收有效问卷（完成3次问卷者）50例。8例因肿瘤进展放弃继续化疗。50例患者中，男性36例，女性14例；年龄50岁-76岁，中位年龄61岁；鳞癌16例，腺癌17例，小细胞癌13例，低分化癌4例；Ⅲ期22例，Ⅳ期28例；有基础疾病者21例（包括高血压、冠心病、慢性支气管炎、支气管哮喘、脑出血、糖尿病等），无基础疾病者29例；化疗后2周期，CR 0例，PR 19例，SD 12例，PD 19例，有效率为38%；化疗后4周期，CR 0例，PR 10例，SD 10例，PD 30例，有效率为20%（其中小细胞肺癌化疗4周期均无进展）。

### 2.2 生活质量得分改变

肺癌患者化疗前后生活质量各领域得分及比较见表 1。与化疗前相比，化疗后2周期功能领域中角色、社会功能得分下降。症状领域中疼痛、恶心呕吐得分上升；单一领域中呼吸困难得分下降，失眠、食欲丧失、便秘、腹泻、经济困难得分上升，总体生活质量得分下降。其中呼吸困难、失眠、食欲丧失条目较前相比有统计学差异（*P* < 0.05）。化疗后4周期功能领域中躯体、角色、情绪、社会功能得分进一步下降；症状领域中疲乏、疼痛、恶心呕吐得分继续上升。单一条目中，呼吸困难、食欲丧失、便秘、经济困难得分上升，整体生活质量得分进一步下降。其中，与化疗后2周期相比，躯体、角色、情绪、社会功能得分下降有统计学差异（*P* < 0.05），恶心呕吐、食欲丧失、便秘、经济困难得分上升有统计学差异（*P* < 0.05）。

**1:** 肺癌患者化疗不同时期EORTC QLQ-C30各领域得分比较（Mean±SD） Compare of the scores for EORTC QLQ-C30 in different periods of chemotherapy (Mean±SD)

	Baseline	After 2 cycles	After 4 cycles	*F*	*P*
Functioning scales					
Physical	75.10±11.49	73.11±11.82	65.40±16.96^△※^	7.015	0.001
Role	66.00±23.00	60.66±19.00	50.00±21.84^△※^	7.680	0.001
Emotion	63.32±24.11	61.66±21.24	47.63±18.53^△^	8.090	0.001
Cognitive	60.99±30.23	64.99±22.14	65.00±19.70	0.048	0.064
Social	63.66±23.75	55.67±18.64	41.65±13.16^△※^	20.11	0.001
Symptom scales					
Fatigue	50.72±24.08	49.71±21.22	57.36±18.57	1.883	0.156
Pain	25.80±20.09	31.67±21.09	38.67±22.41^※^	5.108	0.020
Nausea and vomiting	23.52±17.45	25.42±16.43	34.99±15.92^△※^	9.050	0.001
Specific items reflecting symptoms and economic conditions					
Dyspnoea	50.67±34.51	37.33±26.65^*^	40.66±25.47	2.836	0.062
Insomnia	35.78±30.06	46.33±25.50^*^	46.67±24.30^※^	3.493	0.033
Appetite loss	37.28±26.36	47.65±27.32^*^	68.68±23.73^△※^	16.44	0.003
Constipation	29.33±21.23	37.66±28.59	46.00±30.43^△※^	3.453	0.043
Diarrhea	32.92±23.65	35.00±25.85	34.92±26.47	0.182	0.785
Financial impact	39.83±30.60	42.26±32.23	55.99±33.52^△※^	3.776	0.031
Global quality of life	58.65±21.77	54.32±16.62	45.69±14.22^※^	7.113	0.001
^*^Compare with before chemotherapy and after 2 cycles, *P* < 0.05; ^△^Compare with after 2 cycles and after 4 cycles, *P* < 0.05; ^※^Compare with before chemotherapy and after 4 cycles, *P* < 0.05.					

### 2.3 焦虑情绪改变

化疗前、化疗后2周期、化疗后4周期，50例患者中有焦虑情绪的分别为28例（56%）、40例（80%）、36例（72%）。SAS得分分别为49.54±5.64、52.48±6.10、54.82±6.55。化疗后2周期焦虑得分与化疗前相比有统计学差异（*P* < 0.05），化疗后4周期与化疗前相比亦有统计学差异（*P* < 0.05），与化疗后2周期相比无统计学差异（图 1）。

**1:**
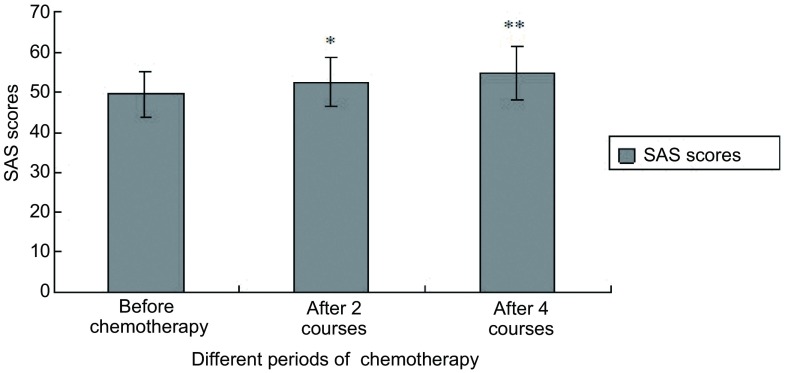
肺癌患者化疗前、化疗后2周期、化疗后4周期的SAS得分比较。^*^化疗前与化疗后2周期相比，*P* < 0.05；^**^化疗前与化疗后4周期相比，*P* < 0.05。 Compare of the scores for SAS before chemotherapy, after 2 courses of chemotherapy, after 4 courses of chemotherapy. ^*^Compare with before chemotherapy and after 2 cycles, *P* < 0.05; ^**^Compare with before chemotherapy and after 4 cycles, *P* < 0.05.

### 2.4 SAS得分与性别、年龄、BMI、KPS、分期、病理类型、有无基础病、文化程度、有无转移的关系

化疗前后SAS得分与性别、年龄、BMI、病理类型、分期及有无远处转移无明显相关性（*P* > 0.05）。化疗前、化疗后2周期焦虑症状得分与KPS无明显相关性，化疗后4周期SAS得分与KPS呈负相关（*P* < 0.05）。化疗后2周期有基础疾病的患者SAS得分高于无基础疾病的患者，差异有统计学意义（*P* < 0.05）（表 2、表 3）。

**2:** 肺癌患者SAS得分与年龄、BMI、KPS、肺癌分期相关关系（*Pearson*线性相关） The correlation between SAS scores and age, BMI, KPS and staging (*Pearson* linear correlation)

	Before chemotherapy	After 2 cycles	After 4 cycles
Age	0.104	0.082	0.080
BMI	0.008	-0.129	-0.249
KPS	-0.107	-0.142	-0.294^*^
Staging	0.056	0.124	0.056
^*^P < 0.05.			

**3:** 肺癌患者化疗前后不同性别、病理类型、有无转移、文化程度、有无基础病之间的SAS得分（*t*检验） The SAS scores of different gender, pathological types, metastasis, educational background and disease history of lung cancer patients before and after chemotherapy (*t*-test)

	Before chemotherapy	*t*	*P*	After 2 cycles	*t*	*P*	After 4 cycles	*t*	*P*
	SAS scores			SAS scores			SAS scores		
Gender		-0.148	0.883		-0.558	0.675		0.124	0.901
Female	48.93±7.332			52.79±8.559			54.57±8.671		
Male	49.19±4.944			51.92±5.623			54.31±5.932		
Histological types		-0.940	0.352		-0.558	0.559		-0.145	0.885
SCLC	50.38±4.629			53.08±6.474			54.62±5.284		
NSCLC	54.30±7.421			51.92±5.623			54.30±7.421		
Metastasis		1.270	0.210		0.007	0.995		0.718	0.476
Yes	50.17±4.770			52.17±4.896			53.67±5.147		
No	48.15±6.265			52.15±7.786			55.04±7.942		
Educational background		-0.911	0.367		0.664	0.510		0.685	0.497
≤9 years	58.60±0.977			51.78±6.774			53.97±6.897		
> 9 years	50.14±1.340			53.14±5.829			55.43±6.345		
Disease history		-0.886	1.388		-2.394	0.675		-1.287	0.204
No	48.68±5.940			51.84±6.560			54.30±7.421		
Yes	50.38±4.629			53.08±6.474^*^			54.62±5.284		
^*^P < 0.05. SCLC: small cell lung cancer; NSCLC: non-small cell lung cancer.									

### 2.5 SAS得分与EORTC QLQ-C30各领域得分关系

化疗前，焦虑症状与失眠轻度相关（*P* < 0.05）；化疗后2周期焦虑症状与疲乏、呼吸困难轻度相关（*P* < 0.05）。化疗后4周期，焦虑症状与疲乏、失眠明显相关（*P* < 0.01），与便秘轻度相关（*P* < 0.05）（表 4）。

**4:** 肺癌患者化疗前后SAS得分与生活质量各条目得分的关系（*Pearson*线性相关分析） The correlation between SAS scores and items of EORTC QLQ-C30 before and after chemotherapy of lung cancer patients (*Pearson* linear correlation)

	Before chemotherapy	After 2 cycles	After 4 cycles
Functioning scales			
Physical	0.047	-0.006	0.161
Role	0.047	-0.044	-0.164
Emotion	0.095	-0.196	-0.078
Cognitive	-0.057	-0.183	-0.066
Social	0.020	-0.252	0.148
Symptom scales			
Fatigue	0.157	0.341^*^	0.417^**^
Pain	-0.003	-0.003	0.102
Nausea and vomiting	0.001	-0.036	0.158
Specific items reflecting symptoms and economic conditions			
Dyspnoea	0.227	0.436^*^	0.049
Insomnia	0.318^*^	0.241	0.362^**^
Appetite loss	0.117	0.066	-0.037
Constipation	-0.043	0.196	0.285^*^
Diarrhea	-0.062	-0.138	0.173
Financial impact	-0.011	0.091	0.341^*^
Global quality of life	-0.154	-0.232	0.075
^*^P < 0.05; ^**^P < 0.01.			

## 3 讨论

生活质量是一种日常生活的状态，包括完成日常生活的能力，比如躯体活动、心理活动以及社会生活和患者对自己功能的满意程度以及对疾病的控制力^[4]^。生活质量在癌症的治疗过程中逐渐显示出了它的重要性，成为重要的评价治疗疗效的标准^[5]^。

化疗是治疗肺癌的最有效方法之一，但会对机体产生一定的不良反应。随着化疗的进行，患者呼吸困难改善明显，但疼痛、食欲不振、失眠、恶心呕吐、财务困难逐渐加重，社会功能下降。其中，食欲不振、恶心呕吐与癌症本身及化疗药物、止疼药物的副作用相关。化疗药物本身的不良反应及患者对化疗过程的情绪反应都会导致睡眠质量下降^[6]^。肺癌患者的身体状况和反复住院治疗妨碍患者的家庭生活和社交活动，社会价值受到影响。肺癌患者的经济支出与疾病严重程度成正相关^[7]^，昂贵的医疗费用与患者有限的经济支付能力成为治疗过程中较为突出的一对矛盾。

焦虑是指一种缺乏明显客观原因的内心不安或无根据的恐惧^[8-10]^。肺癌患者中，焦虑是最常出现的心理特征^[11]^，但也很容易被医务人员所忽视和低估。过度的焦虑对癌症的转归及生活质量有着极为不利的影响。本研究发现，随着化疗的进行，癌症患者焦虑情绪也逐步加重，考虑与化疗过程中症状加重、体力状态变差、预后生存期短及经济情况恶化有关^[10]^。

肺癌患者焦虑症状与年龄、性别、BMI、病理类型、分期、文化程度、有无转移无明显相关性。这可能是年龄、性别、职业所造成的心理差异已为癌症的痛苦所掩盖，同时不排除由于本研究病例过少所导致。Linden等^[12]^对10, 153例癌症患者的研究表明焦虑情绪在年轻、女性患者中更为普遍，因年轻患者是家庭的重要支柱，诊断明确后情绪波动大，易出现焦虑等不良心理状态，而女性较男性更倾向于表达自己的情绪。本研究发现化疗后4周期焦虑症状与KPS评分呈负相关。低KPS得分患者，日常生活需要家人协助，对自己的能力产生怀疑，焦虑症状更加严重。有基础疾病的患者需时刻注意自己的基础病病情，生活习惯、饮食方面不同于健康人，且大部分需要长期服用药物治疗，在患肺癌之前就存在焦虑情绪，而肺癌的确诊、化疗更添加了患者的焦虑。本研究未发现文化程度对患者焦虑有明显影响，但Byrne^[13]^发现文化程度高、不吸烟、已婚的癌症患者较文化程度低的吸烟、单身患者焦虑症状明显，可能是在看待问题的态度上，文化程度高的要豁达些，文化低的要简单些。

肺癌患者往往承受很大的心理压力，尤其是中晚期肺癌患者出现呼吸困难、疲乏等症状，使得患者的心理压力更大，加重了焦虑情绪，导致睡眠结构的改变^[14]^，Sela^[15]^也发现有睡眠障碍的患者往往同时合并有焦虑和疲乏症状。焦虑会加重患者的呼吸困难，而加重的呼吸困难反之会加剧患者的焦虑症状，形成一个正反馈回路^[16, 17]^。疲劳、呼吸困难和焦虑之间存在较强的联系，提示存在症候群^[18]^，对患者日常生活产生种种限制。

综上所述，随着化疗周期的增加，肺癌患者部分症状改善，但生活质量较前下降，焦虑情绪较前增加，影响癌症的转归。因此，医务人员要特别注意化疗引起的不良反应并及时采取措施，不仅对患者进行药物治疗，应更加注重患者的心理状态，改善患者的生活质量，给予正确、有效的心理咨询及情感支持。
